# [Corrigendum] Thymoquinone inhibits epithelial‑mesenchymal transition in prostate cancer cells by negatively regulating the TGF‑β/Smad2/3 signaling pathway

**DOI:** 10.3892/or.2023.8491

**Published:** 2023-02-02

**Authors:** Bo Kou, Wei Liu, Wei Zhao, Peng Duan, Yang Yang, Qiuyue Yi, Fengwei Guo, Jianpeng Li, Jinsong Zhou, Qingshan Kou

Oncol Rep 38: 3592–3598, 2017; DOI: 10.3892/or.2017.6012

After the publication of the article, an interested reader drew to the authors' attention that the Du145 ‘Control’ migration panel in Fig. 2C appeared to overlap with the Du145 ‘Control’ invasion panel in Fig. 5A; furthermore, two of the Du145 panels in Fig. 5A also appeared to overlap. The authors have consulted their original data, and realize that these figures were inadvertently assembled incorrectly.

The corrected versions of Figs. 2 and 5, incorporating the correct data for the Du145 ‘Control’ panel in Fig. 2C, and the TQ-/TGF-β OE- invasion and migration panels, and the TQ+/TGF-β OE+ migration panel, in Fig. 5A, are shown on the next page. These further corrections do not grossly affect the results or the conclusions reported in this work. The authors all agree to this Corrigendum, and are grateful to the Editor of *Oncology Reports* for granting them the opportunity to correct the errors that were made during the assembly of these figures. Lastly, the authors apologize to the readership for any inconvenience these errors may have caused.

## Figures and Tables

**Figure 2.**
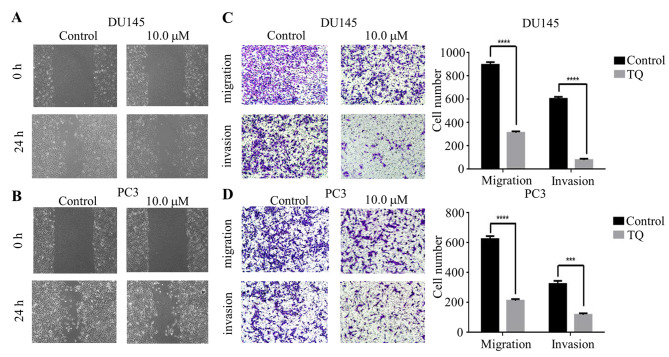
Thymoquinone (TQ) inhibits the migratory and invasive capacity of prostate cancer DU145 and PC3 cells. The width of scratches was detected with or without thymoquinone treatment at 0 and 24 h in prostate cancer (A) DU145 and (B) PC3 cells. (C and D) In addition, using Transwell migration and Matrigel invasion assays, the numbers of migrated and invaded (C) DU145 and (D) PC3 cells treated with 10 µM thymoquinone/chamber were determined and compared with these numbers in the control group from three independent experiments (***P<0.001 and ****P<0.0001).

**Figure 5.**
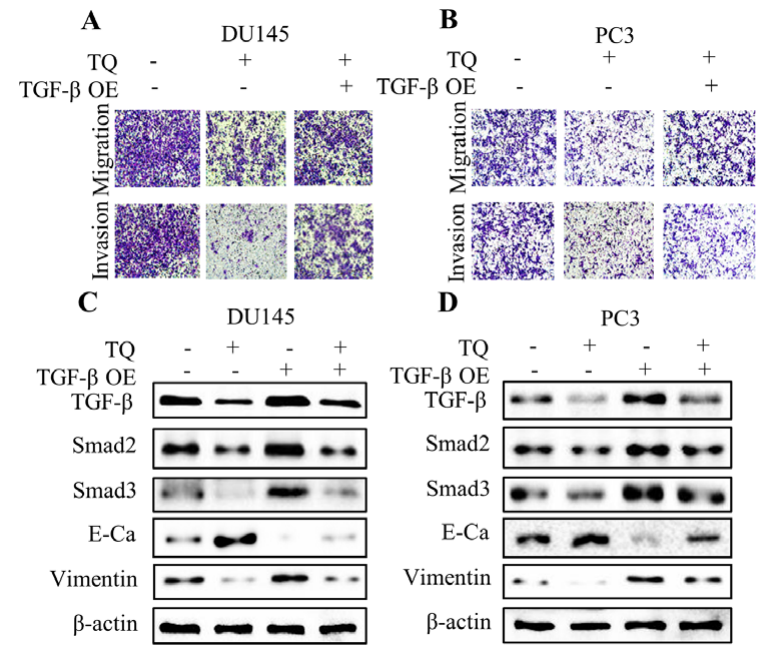
- Overexpression (OE) of TGF-β attenuates the antitumor capacity of thymoquinone (TQ) in prostate cancer cells. Transwell migration and Matrigel invasion assays were performed on (A) DU145 and (B) PC3 cells following combination treatment of TQ and TGF-β overexpression. Five random fields were chosen and visualized by microscopy to assess the cell migratory and invasive capacity. Western blotting was performed to evaluate the levels of TGF-β, Smad2, Smad3, E-cadherin (E-Ca), vimentin and β-actin protein upon various treatments in the (C) DU145 and (D) PC3 cells. Representative protein bands of three experiments are shown.

